# Supplementary material for: Comparative genomics provides new insights into the diversity, physiology, and sexuality of the only industrially exploited tremellomycete: Phaffia rhodozyma
Source: BMC Genomics. 2016 Nov 9;17:901. doi: 10.1186/s12864-016-3244-7 (PMC5103461; doi:10.1186/s12864-016-3244-7)
Supplement: Additional file 6: — List of orphan genes with links to PFAM (related to Additional file 1: Table S1). (ZIP 1428 kb) [file 12864_2016_3244_MOESM6_ESM.zip › BLAST_HTML_FTR/G01533_P.html]

BLAST Search Results


```
BLASTP 2.2.27+


Reference:
Stephen F. Altschul, Thomas L. Madden, Alejandro A. Schäffer,
Jinghui Zhang, Zheng Zhang, Webb Miller, and David J. Lipman (1997),
"Gapped BLAST and PSI-BLAST: a new generation of protein database
search programs", Nucleic Acids Res. 25:3389-3402.


Reference for
composition-based statistics:
Alejandro A. Schäffer, L. Aravind, Thomas L. Madden, Sergei
Shavirin, John L. Spouge, Yuri I. Wolf, Eugene V. Koonin, and
Stephen F. Altschul (2001), "Improving the accuracy of PSI-BLAST
protein database searches with composition-based statistics and
other refinements", Nucleic Acids Res. 29:2994-3005.


Database: nr
           71,551,133 sequences; 26,053,659,533 total letters


Query= G01533_P

Length=758
                                                                      Score     E
Sequences producing significant alignments:                          (Bits)  Value

emb|CED83061.1|  hypothetical protein [Xanthophyllomyces dendrorh...  1514    0.0  
ref|XP_010480102.1|  PREDICTED: serine/threonine-protein kinase B...  42.7    2.1  
ref|XP_010501177.1|  PREDICTED: serine/threonine-protein kinase B...  41.6    4.6  


 >emb|CED83061.1| hypothetical protein [Xanthophyllomyces dendrorhous]
Length=757

 Score = 1514 bits (3920),  Expect = 0.0, Method: Compositional matrix adjust.
 Identities = 757/757 (100%), Positives = 757/757 (100%), Gaps = 0/757 (0%)

Query  1    MTCVVPCKDVDVSESDLAGKITLYGENTSIRPKESSDSSDASSYSFTCPIPARHTSSINI  60
            MTCVVPCKDVDVSESDLAGKITLYGENTSIRPKESSDSSDASSYSFTCPIPARHTSSINI
Sbjct  1    MTCVVPCKDVDVSESDLAGKITLYGENTSIRPKESSDSSDASSYSFTCPIPARHTSSINI  60

Query  61   VDVDDNDDEDDIGSWDDRQFGVFRKLPFLGVPDDLKPVISNPIDIPKDQFLRAMPASSDS  120
            VDVDDNDDEDDIGSWDDRQFGVFRKLPFLGVPDDLKPVISNPIDIPKDQFLRAMPASSDS
Sbjct  61   VDVDDNDDEDDIGSWDDRQFGVFRKLPFLGVPDDLKPVISNPIDIPKDQFLRAMPASSDS  120

Query  121  TILENIVIGGPKVSVTTCSSISNDISGGPEFSMVDETNEVLDDELNFTSGSLPSPSFNGC  180
            TILENIVIGGPKVSVTTCSSISNDISGGPEFSMVDETNEVLDDELNFTSGSLPSPSFNGC
Sbjct  121  TILENIVIGGPKVSVTTCSSISNDISGGPEFSMVDETNEVLDDELNFTSGSLPSPSFNGC  180

Query  181  AQVPSVSVICLDSDSRCPVTDTRERSGEKEVEGRPCWLDISLDNGDISAGGDTRIDVAEK  240
            AQVPSVSVICLDSDSRCPVTDTRERSGEKEVEGRPCWLDISLDNGDISAGGDTRIDVAEK
Sbjct  181  AQVPSVSVICLDSDSRCPVTDTRERSGEKEVEGRPCWLDISLDNGDISAGGDTRIDVAEK  240

Query  241  VVMIKPSDPLQGISSLSNTSKYISKRFGSSGSPCNNPFSSPAVPTTTARNESLGSSSSLN  300
            VVMIKPSDPLQGISSLSNTSKYISKRFGSSGSPCNNPFSSPAVPTTTARNESLGSSSSLN
Sbjct  241  VVMIKPSDPLQGISSLSNTSKYISKRFGSSGSPCNNPFSSPAVPTTTARNESLGSSSSLN  300

Query  301  LNTLNRSTPSLTADLSSSFFSSPSLADLTNPNSPLIDVDDQSHSSPLKSSRRNILFTNVK  360
            LNTLNRSTPSLTADLSSSFFSSPSLADLTNPNSPLIDVDDQSHSSPLKSSRRNILFTNVK
Sbjct  301  LNTLNRSTPSLTADLSSSFFSSPSLADLTNPNSPLIDVDDQSHSSPLKSSRRNILFTNVK  360

Query  361  KVDMVPPAPLPPPTVSTVQDSDSELEFGTEPDVDSIVNVPFSQSRRVPFPYSAPRSSLGN  420
            KVDMVPPAPLPPPTVSTVQDSDSELEFGTEPDVDSIVNVPFSQSRRVPFPYSAPRSSLGN
Sbjct  361  KVDMVPPAPLPPPTVSTVQDSDSELEFGTEPDVDSIVNVPFSQSRRVPFPYSAPRSSLGN  420

Query  421  RSVASLSYPKKPEPSIDSIFGVPKLDDKIACATDNSDSRMKWMDRTVPRAPRWSKRRKMT  480
            RSVASLSYPKKPEPSIDSIFGVPKLDDKIACATDNSDSRMKWMDRTVPRAPRWSKRRKMT
Sbjct  421  RSVASLSYPKKPEPSIDSIFGVPKLDDKIACATDNSDSRMKWMDRTVPRAPRWSKRRKMT  480

Query  481  FGDSDIDSPTARKHRLRQEEYGNLELNFTAWHGKLKHEVRDLKSTPPSRIDLANNKSRFD  540
            FGDSDIDSPTARKHRLRQEEYGNLELNFTAWHGKLKHEVRDLKSTPPSRIDLANNKSRFD
Sbjct  481  FGDSDIDSPTARKHRLRQEEYGNLELNFTAWHGKLKHEVRDLKSTPPSRIDLANNKSRFD  540

Query  541  ADCSIQPTNSLTTKSSLICPSPQMNRPRKRLASPSSPACASVSSPLTQACLAKEKNRSSQ  600
            ADCSIQPTNSLTTKSSLICPSPQMNRPRKRLASPSSPACASVSSPLTQACLAKEKNRSSQ
Sbjct  541  ADCSIQPTNSLTTKSSLICPSPQMNRPRKRLASPSSPACASVSSPLTQACLAKEKNRSSQ  600

Query  601  AEASLRRGVFDSYSERDNSPRTSEADQIEEEEKMEEEEEQILVDKSLPLPPPSSSPTPCT  660
            AEASLRRGVFDSYSERDNSPRTSEADQIEEEEKMEEEEEQILVDKSLPLPPPSSSPTPCT
Sbjct  601  AEASLRRGVFDSYSERDNSPRTSEADQIEEEEKMEEEEEQILVDKSLPLPPPSSSPTPCT  660

Query  661  PSLSAVSVFSLASCLSCTPSGIKSSLPPASIIQSSANPIEFENDNQTSEDEDSPFMGRLF  720
            PSLSAVSVFSLASCLSCTPSGIKSSLPPASIIQSSANPIEFENDNQTSEDEDSPFMGRLF
Sbjct  661  PSLSAVSVFSLASCLSCTPSGIKSSLPPASIIQSSANPIEFENDNQTSEDEDSPFMGRLF  720

Query  721  TCAYTRSVSPDTSWMKQRQLSVDVPDDEDPLACVDVW  757
            TCAYTRSVSPDTSWMKQRQLSVDVPDDEDPLACVDVW
Sbjct  721  TCAYTRSVSPDTSWMKQRQLSVDVPDDEDPLACVDVW  757


>ref|XP_010480102.1| PREDICTED: serine/threonine-protein kinase BRI1-like 1 [Camelina 
sativa]
Length=1167

 Score = 42.7 bits (99),  Expect = 2.1, Method: Compositional matrix adjust.
 Identities = 52/190 (27%), Positives = 86/190 (45%), Gaps = 22/190 (12%)

Query  10   VDVSESDLAGKITLYGENTSIRPKESSDSS-----DASSYSFTCPIPARHTSSINIVDVD  64
            V+ S + LAGK+  +  ++S++   + D S     +    SF   +PA    S+  +D+ 
Sbjct  156  VNFSNNKLAGKLG-FSPSSSLKSLTTVDLSYNILSEKIPESFISDLPA----SLKYLDLT  210

Query  65   DNDDEDDIGSWDDRQFGVFRKLPFLGVPDDLKPVISNPIDIPKDQFLRAMPASSDSTILE  124
             N+   D   + D  FG+   L FL +  +       PI +P  +FL  +  S ++  L 
Sbjct  211  HNNLSGD---FSDLSFGICGNLTFLSLSQNNISGDKFPITLPNCEFLETLNISRNN--LA  265

Query  125  NIVIGGPKVS----VTTCSSISNDISGG--PEFSMVDETNEVLDDELNFTSGSLPSPSFN  178
              + GG        +   S   N  SG   PE S++ +T EVLD   N  SG LP P F 
Sbjct  266  GKIPGGEYWGGFQNLRHLSLAHNRFSGEIPPELSLLCKTLEVLDLSGNALSGELP-PQFA  324

Query  179  GCAQVPSVSV  188
             C  + ++++
Sbjct  325  ACVSLQNLNL  334


>ref|XP_010501177.1| PREDICTED: serine/threonine-protein kinase BRI1-like 1 isoform 
X1 [Camelina sativa]
 ref|XP_010501178.1| PREDICTED: serine/threonine-protein kinase BRI1-like 1 isoform 
X2 [Camelina sativa]
Length=1169

 Score = 41.6 bits (96),  Expect = 4.6, Method: Compositional matrix adjust.
 Identities = 51/190 (27%), Positives = 85/190 (45%), Gaps = 22/190 (12%)

Query  10   VDVSESDLAGKITLYGENTSIRPKESSDSS-----DASSYSFTCPIPARHTSSINIVDVD  64
            V+ S + LAGK+  +  ++S++   + D S     +    SF    PA    S+  +D+ 
Sbjct  158  VNFSNNKLAGKLG-FAPSSSLKSLTTVDLSYNILSEKIPESFISDFPA----SLKYLDLT  212

Query  65   DNDDEDDIGSWDDRQFGVFRKLPFLGVPDDLKPVISNPIDIPKDQFLRAMPASSDSTILE  124
             N+   D   + D  FG+   L FL +  +       PI +P  ++L  +  S ++  L 
Sbjct  213  HNNLSGD---FSDLSFGICGNLTFLSLSQNNISGDKFPITLPNCEYLETLNISRNN--LA  267

Query  125  NIVIGGPKVS----VTTCSSISNDISGG--PEFSMVDETNEVLDDELNFTSGSLPSPSFN  178
              + GG        +   S   N  SG   PE S++ +T EVLD   N  SG LP P F 
Sbjct  268  GKIPGGEYWGGFQNLKQLSLAHNRFSGEIPPELSLLCKTLEVLDLSGNALSGELP-PQFA  326

Query  179  GCAQVPSVSV  188
             C  + ++++
Sbjct  327  ACVSLQNLNL  336


Lambda      K        H        a         alpha
   0.311    0.128    0.375    0.792     4.96 

Gapped
Lambda      K        H        a         alpha    sigma
   0.267   0.0410    0.140     1.90     42.6     43.6 

Effective search space used: 8791540602214


  Database: nr
    Posted date:  Sep 23, 2015 12:05 AM
  Number of letters in database: 26,053,659,533
  Number of sequences in database:  71,551,133


Matrix: BLOSUM62
Gap Penalties: Existence: 11, Extension: 1
Neighboring words threshold: 11
Window for multiple hits: 40
```
